# Radiotherapy in the treatment of malignant fungating wounds: clinical practice, response rates, and outcome from a tertiary cancer center

**DOI:** 10.1007/s00066-025-02443-7

**Published:** 2025-08-12

**Authors:** Anna Lena Reinking, Martin Leu, Leif Hendrik Dröge, Benedikt Kieslich, Sandra Donath, Markus Anton Schirmer, Stephanie Bendrich, Laura Anna Fischer, David Alexander Ziegler, Hannes Treiber, Enver Aydilek, Raphael Koch, Stefan Rieken, Manuel Guhlich

**Affiliations:** 1 Clinic of Radiotherapy and Radiation Oncology, University Medical Center Göttingen, Göttingen, Germany; 2 Department of Hematology and Medical Oncology, University Medical Center Göttingen, Göttingen, Germany; 3 Clinic of Radiation Oncology and Radiotherapy, Medical University Lausitz—Carl Thiem, Cottbus, Germany; 4 Quality Conferences Office at the Clinical State Registry Baden-Württemberg GmbH, Baden-Württemberg Cancer Registry (BWCR), Stuttgart, Germany

**Keywords:** Malignant fungating wound, Radiotherapy, Radiation, Symptom relief, Palliative medicine, Palliative radiotherapy

## Abstract

**Purpose:**

Malignant fungating wounds (MFW) are a distressing condition caused by aggressive tumor growth infiltrating the skin. Regularly causing pain, exudation, bleeding, edema and odor, they negatively affect the patients’ quality of life (QoL). Radiotherapy (RT) can reduce MFW-associated symptoms and is routinely used in clinical settings, both in curative as well as palliative treatment regimes. However, fundamental data on treatment response, symptom relief and oncological outcomes, as well as potential confounders of treatment response are currently limited.

**Methods:**

We performed a retrospective analysis of patients with MFW who received RT between 01/2000 and 06/2022 at our tertiary cancer center. Achievement of treatment goals, including reduction of pain and tumor mass, cessation of bleeding, and improvement of wound condition, were evaluated. The effect of variables on the achievement of treatment goals were assessed by logistic regression. The effect of parameters on overall survival (OS) were assessed using the Kaplan-Meier plot with log-rank test and Cox regression analysis. Statistically significant (*p*-value < 0.05) confounders were tested in multivariable analyses.

**Results:**

101 patients were included. 69.3% of treatments were in palliative intent, 30.7% in curative intent. Main tumor entities were breast cancer, squamous cell carcinoma of the skin and vulvar carcinoma, accounting for 26.7, 22.8 and 9.9% of patients. Main treated locations were head & neck (38.6%), breast/chest wall (29.7%) and genitals (9.9%). Main treated areas were primary tumor (52.5%) and metastasis (22.8%). Concurrent systemic therapy was administered in 32.7%. The predefined therapy goal was achieved in 85% of patients. Median overall survival was 7.8 months. Concurrent systemic therapy was statistically significant associated with achieving the therapy goal [logistic regression; HR 8.45 (95% CI: 1.06–67.37, *p* = 0.04)]. Concurrent systemic therapy, lower CCI and achieving the therapy related goal were significantly associated with higher overall survival. Overall toxicity was low.

**Conclusion:**

RT for MFW is a highly effective treatment option, resulting in very high local tumor regression rates. It therefore reduces the numerous negative QoL-affecting consequences for the patients, which often present in a palliative state. Concurrent systemic therapy can be a prognostically relevant treatment option.

**Supplementary Information:**

The online version of this article (10.1007/s00066-025-02443-7) contains supplementary material, which is available to authorized users.

## Introduction

A key characteristic of malignant tumors is their ability to infiltrate surrounding tissue. This aspect is important in the development of malignant wounds, which are caused by the infiltration of tumor cells into the skin, its blood- and lymphatic vessels. The results include reduced blood flow to the tissue, the formation of edema, and ultimately, tissue necrosis. Together with the proliferation of malignant cells, this leads to the formation of malignant wounds [1, 2]. These wounds can be described as malignant fungating wounds (MFW), ulcerating cancer, malignant wounds or ulcerating cancer wounds. They result from a variety of malignancies: Primary skin tumors such as squamous cell carcinoma, basal cell carcinoma, and malignant melanoma may ulcerate. Alternatively, malignant wounds can be caused by internal primary tumors (such as breast cancer, head and neck cancer, or sarcoma), by metastases and/or lymph node metastases, when these infiltrate the skin. Another possibility is the development from cutaneous metastases [3]. In very rare cases, chronic wounds with a long duration such as venous leg ulcers, pressure ulcers or scars and especially burn scars, can undergo a malignant transformation (Marjorlin’s ulcer) [4].

Published studies estimate the prevalence of MFW in tumor patients at 6.6 to 14.5% [1, 5]. Some patients have multiple MFW at different locations. The most common site is breast (49%), followed by neck (21%), thorax (18%), extremities (17%), genitals (17%), and head (13%) [1].

Whereas symptoms of MFW vary widely, pain is the most common symptom, affecting 31% of the patients [6]. Pain can be inflammatory, neuropathic or a combination of the two. In 24% of patients, the overall burden of the tumor interferes with activities of daily living, such as dressing or mobility. About 19% of patients with ulcerated wounds suffer from esthetic distress, which can lead to feelings of shame and social isolation. Other common symptoms are serous or purulent exudates, which occur in 15% of wounds. Unpleasant odors occur in 10% of wounds and are described as foul, fishy or similar to the smell of rotting meat [6]. Bleeding, both from small veins and capillaries and from arteries, and itching may occur [7]. MFW do not heal spontaneously but continue to deteriorate over time without antineoplastic treatment (such as chemotherapy (CT), radiotherapy (RT), antihormone therapy, surgery or immunotherapy) due to their malignant nature [3].

Even though data concerning its impact is scarce, local RT is an established clinical treatment option for MFW and consideration of RT is advised by both the German Guideline Program in Oncology as well as the NCCN Guidelines for palliative care [8, 9]. RT has been shown to reduce exudation, edema and the risk of bleeding as well as to reduce disfiguring or difficult-to-care-for tumor manifestations [10].

There are few prospective and retrospective studies mainly focused on malignant wounds of breast cancer as well as a variety of case reports [10–13]. However, overall data of this important treatment option of RT in literature remains underrepresented, details concerning its palliative effectiveness, RT dose and RT technique schemes remain unclarified. In order to broaden the fundamental data, we conducted this retrospective study.

## Patients and methods

### Methods

This single-center retrospective study includes patients treated at the Department of Radiotherapy and Radiooncology, University Medical Center Göttingen, Germany, from 01/2000 to 06/2022. Patients and their corresponding diagnoses were identified by a systematic keyword search for “malignant wound”, “malignant fungating wound”, “ulcerating cancer” “malignant wound” or “ulcerating cancer wound” and supplemented by a review of radiotherapy wound documentation. Data were collected from physical patient records and the radiotherapy treatment planning system (Varian Eclipse, version 15.6, Varian Medical Systems, Palo Alto, USA). The follow-up of the patients was evaluated using the internal data processing systems of the hospital (ixserv.4, version R20.3, ix.mid software technology, Cologne, Germany) and ONKOSTAR (version 2.11.1.1, IT-Choice Software AG, Karlsruhe, Germany). Follow-up screening included reviewing all accessible radiologic data. In the case of post treatment CT or MRI data of the treated area, diagnostic images were fused with the RT treatment planning CT in order to assess treatment response and/or local recurrence (software: Varian Eclipse, version 15.6, Varian Medical Systems, Palo Alto, USA). The study was conducted in accordance with the tenets of the Declaration of Helsinki and was approved by the Ethics Committee of the University Medical Center Göttingen (protocol code: 22/11/22; approval date: November 16, 2022). Data were analyzed using the software SPSS (v. 26) and R (v. 4.0.2) with the “KMWin” (Kaplan–Meier for Windows) plugin [14]. Survival statistics were evaluated using the Kaplan-Meier-estimator. Treatment response, potential adverse events and laboratory results were documented at least once a week during the RT course. They were reviewed by experienced radiation oncologists at consultant level. Survival times were compared using log-rank tests. Univariable and multivariable Cox regression was used to assess the effect of variables on overall survival, and univariable log regression was used for achievement of the therapy goal. *P* values < 0.05 were considered statistically significant. Univariably significant variables were tested multivariably.

### Endpoints

Primary endpoint was achievement of the treatment-related therapy goal, which was defined due to the status at RT initiation: In the case of threatening ulceration as an indication for RT (*n* = 3), the treatment goal was to prevent ulceration. When ulceration was present (*n* = 98), the therapy goal was a substantial regression of the symptoms caused by the tumor wound. Namely, this included a subjective reduction of pain and/or a bleeding stop and/or a reduction in tumor mass and/or an improvement in wound conditions. Improvement of wound condition was based on the Haisfield-Wolfe and Baxendale-Cox stages of malignant wounds as reduction in wound depth and area, reduction in infiltrated skin layers, change in wound color from red/pink/yellow to red/pink, drying of moist wounds, and reduction in wound exudate [15].

While a substantial amount of patients achieved a variety of these goals, the presence of one of these criteria was sufficient to assess the treatment goal as achieved. The presence of tumor progression or an increase in ulceration during RT led to the determination that the treatment goal had not been achieved (even though some of the above-mentioned goals might have been achieved). Definition of treatment goal achievement was set at 8 weeks after RT completion.

Secondary endpoints were overall survival (OS), time to progression (TTP), progression-free survival (PFS) and therapy related adverse events. Concerning OS, death from any cause was counted as event. Time to progression (TTP) was defined as the time difference between the start of RT and the occurrence of local progression or local recurrence. Progression-free survival (PFS) was defined as the time from the start of RT to the first occurrence of local or distant progression or relapse or death of the patient. Toxicity was scored according to Common Terminology Criteria for Adverse Events (Version 5.0) [16].

### Patients

**Fig. 1:**
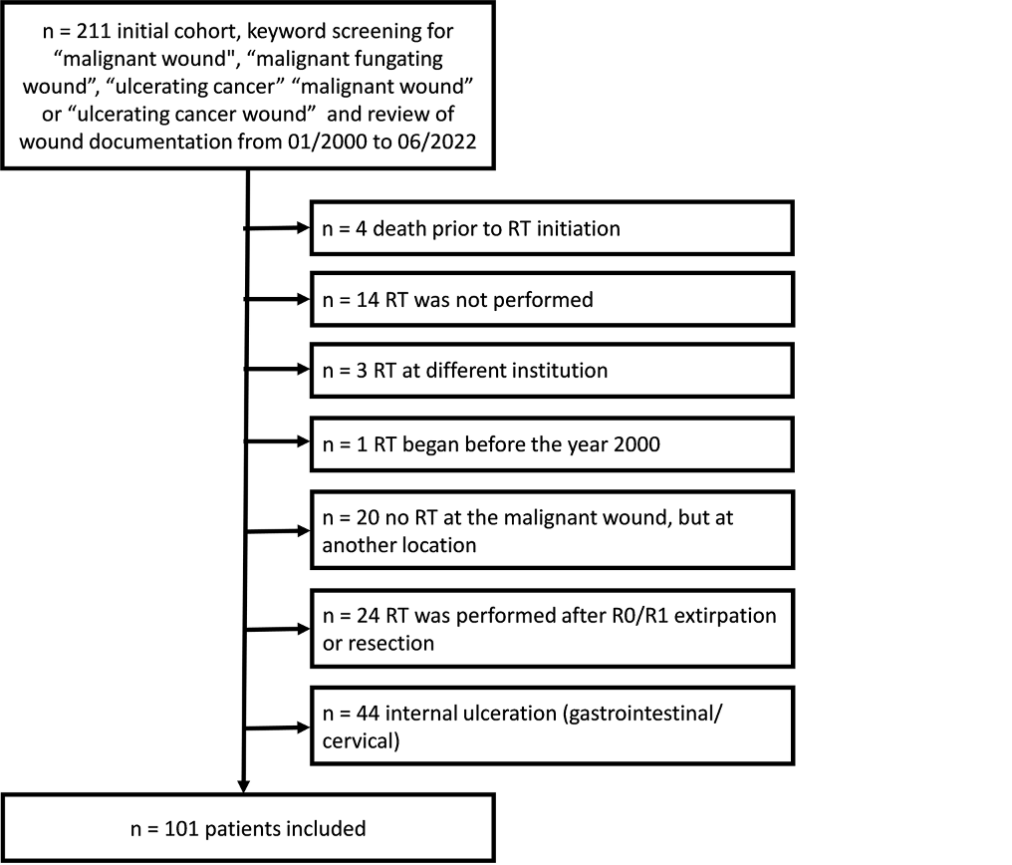
Patient selection flowchart. Initial screening for the keywords “malignant wound”, “malignant fungating wound”, “ulcerating cancer” “malignant wound” or “ulcerating cancer wound” in patient record systems and review of wound documentation from 01/2000 to 06/2022

A total of 101 patients met the inclusion criteria for analysis. For patients receiving RT for MFW in different locations, the chronologically first MFW was included in the study. Details of patient selection are shown in Fig. 1.

Patients age ranged from 28 to 95 years, whereas 65.3% (*n* = 66) of patients were female. Prior to the start of RT, all patients had histologically confirmed tumors. Tumor entities were grouped into thoracic malignancies, skin tumors, pelvic malignancies, Head and Neck tumors, non-Hodgkin’s lymphoma and sarcoma. Thoracic malignancies were the most common at 30.7% (*n* = 31), with breast malignancies accounting for 26.7% (*n* = 27). Of the skin tumors, which represented a total of 28% (*n* = 28), cutaneous squamous cell carcinoma was the most frequent accounting for 22.8% (*n* = 23). Comorbidities were assessed using the Charlson Comorbidity Index (CCI). CCI was between 7 and 10 points in 52.5% of patients. Please refer to Table 1 for the baseline characteristics of the patients and the tumor entities.

**Table 1:** Baseline patient characteristics and tumor entities assigned by anatomical region.

**Patients characteristics**	***n*** **(%)**	**Median (min–max)**
*Patients*	101 (100)	–
*Sex*		
Male	35 (34.7)	–
Female	66 (65.3)	–
*Age, in years*	–	71 (28–95)
*Charlson Comorbidity Index, in points*	–	7 (2–13)
1–3	7 (6.9)	–
4–6	35 (34.7)	–
7–10	53 (52.5)	–
11–13	6 (5.9)	–
**Tumor entities**	***n*** **(%)**	**–**
*Thoracic malignancies*	**31 (30.7)**	**–**
Breast Cancer	27 (26.7)	–
NSCLC	3 (3.0)	–
Pleuramesothelioma	1 (1.0)	–
*Skin tumors*	**28 (27.2)**	**–**
Cutaneous SCC	23 (22.8)	–
Merkel cell carcinoma	2 (2.0)	–
Malignant melanoma	2 (2.0)	–
Basal cell carcinoma	1 (1.0)	–
*Pelvic malignancies*	**19 (18.8)**	**–**
Vulvar carcinoma	10 (9.9)	–
Anal carcinoma	4 (4.0)	–
Endometrial carcinoma	1 (1.0)	–
Ovarian carcinoma	1 (1.0)	–
Rectal carcinoma	1 (1.0)	–
Urinary bladder carcinoma	1 (1.0)	–
Renal cell carcinoma	1 (1.0)	–
*Non-Hodgkin’s lymphoma*	**10 (9.9)**	**–**
Mycosis fungoides	6 (5.9)	–
Other non-Hodgkin’s Lymphoma*	4 (4.0)	–
*Head and Neck tumors*	**8 (7.9)**	**–**
Hypopharyngeal carcinoma	4 (4.0)	–
Carcinoma of the base of the tongue	2 (2.0)	–
Oropharyngeal carcinoma	1 (1.0)	–
Tonsillar carcinoma	1 (1.0)	–
*Sarcoma*#	**5 (5.0)**	**–**

*NSCLC* non-small cell lung cancer, *SCC* squamous cell carcinoma

* *n* = 4 Non-Hodgkin’s Lymphoma: centroblastic lymphoma, lymphoblastic B‑cell lymphoma, follicular lymphoma, marginal zone lymphoma

# *n* = 5 Sarcoma: Cutaneous angiosarcoma, Kaposi’s sarcoma, undifferentiated dermal sarcoma, osteosarcoma of osteoblastic type

**Fig. 2:**
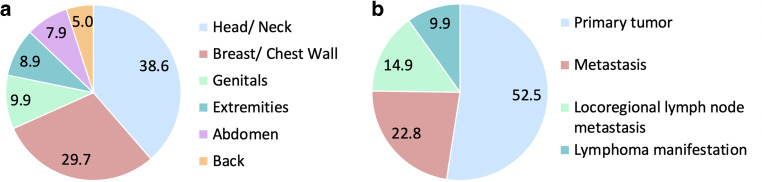
**a** Pie chart of the location of the treated MFW (*n* = 101). **b** Origins of the 101 treated MFW. Numbers are in percentage terms

Malignant wounds were analyzed by location. The most common site were the head and neck with 38.6% (*n* = 39). This was followed by the breast and chest wall with 29.7% (*n* = 30). The genitals accounted for 9.9% (*n* = 10) of ulcerated wounds, the extremities 8.9% (*n* = 9), the abdomen 7.9% (*n* = 8) and the back 5.0% (*n* = 5). Of the malignant wounds, 52.5% (*n* = 53) originated directly from a primary tumor, with locoregional recurrences also counted as primary tumors. Metastases were the cause of a malignant wound in 22.8% (*n* = 23). Locoregional lymph node metastases accounted for 14.9% (*n* = 15) and lymphoma manifestations of NHL occurred in 9.9% (*n* = 10). Please refer to Fig. 2.

In total, *n* = 70 (69.3%) of the patients underwent a palliative treatment concept, *n* = 31 (30.7%) patients received curatively intended RT. A flap was used in 70% (*n* = 67) of the 96 patients who received teletherapy. Flaps thickness was 1.0 cm in 97% (*n* = 65) of the cases and 0.5 cm in 3% (*n* = 2). 21 patients (20.8%) had received prior RT to the site of the current RT. Regarding the current RT course, 94% (*n* = 95) of patients received at least 80% of the planned total dose. 10.9% of patients (*n* = 11) stopped RT due to different reasons: a decline in general condition (*n* = 5), the patient’s will (*n* = 4) or treatment-related side effects (*n* = 2). Concerning the patients who discontinued RT due to adverse events, one patient had grade 3 radiation dermatitis, grade 1 nausea and grade 1 skin hyperpigmentation. The other patient experienced grade 2 radiation dermatitis, grade 2 oral mucositis, grade 1 dysphagia, grade 1 skin hyperpigmentation and grade 1 lymphedema. Please refer to Table 2 for treatment and RT details.

**Table 2:** RT details for a total of *n* = 101 patients.

Treatment characteristics	*n* (%)	Median (min–max)
*Therapy concept*		
Definitive RT	17 (16.8)	–
Definitive radiochemotherapy	10 (9.9)	–
Palliative RT	63 (62.4)	–
Palliative radiochemotherapy	7 (6.9)	–
Neoadjuvant radiochemotherapy	3 (3.0)	–
Neoadjuvant RT	1 (1.0)	–
*RT technique*		
3DcRT	56 (55.4)	–
VMAT	17 (16.8)	–
Electron Standing Field	11 (10.9)	–
IMRT	8 (7.9)	–
Brachytherapy	5 (5.0)	–
VMAT + Brachytherapy	1 (1.0)	–
VMAT + 3DcRT	1 (1.0)	–
IMRT + VMAT	1 (1.0)	–
Electron Standing Field + 3DcRT	1 (1.0)	–
*When Teletherapy (n* *=* 96*): Flap used	67 (69.8)	–
Flap thickness 1.0 cm	65 (97.0)	–
Flap thickness 0.5 cm	2 (3.0)	–
*EQD2, in Gy (a/ß* *=* *10)*	–	48.75 (4.0–80.1)
*Total dose, in Gy*	–	45.0 (4.0–78.0)
*Single dose, Gy*	–	2.0 (1.5–6.0)
*Course of RT*		
Applied dose ≥ 80% of planned dose	95 (94.0)	–
Applied dose < 80% of planned dose	6 (5.9)	–
*CTV size*^*#*^* (n* *=* *61), in ccm*	–	391.6 (14.8–3670.5)
*PTV size*^*$*^* (n* *=* *68), in ccm*	–	842.6 (55.0–5615.3)

*RT* radiotherapy, *VMAT* volumetric modulated arc therapy, *IMRT* intensity modulated RT, *3DcRT* 3D conformal RT, *CTV* clinical target volume, *PTV* planning target volume, *EQD2* equivalent dose in 2‑Gy fractions, *ccm* = cubic centimetre

* Subgroup analysis: only patients who received external beam RT were taken into account for flap evaluation (*n* = 96)

# CTV data available for 61 patients

$ PTV data only for 68 patients

A total of 74 patients experienced RT-related adverse events that were graded according to the Common Terminology Criteria for Adverse Events (version 5.0) [16]. Of these patients, 28 (27.7%) had the worst adverse event classified as grade 1, 35 (34.7%) as grade 2, and 11 (10.9%) as grade 3.

The most common adverse event at all as well as the most common grade 3 toxicity was radiation dermatitis, which was observed in a total of 66 patients (65.3%). For details concerning RT-related toxicities, please refer to the supplemental material, supplemental Table 1.

## Results

### Achievement of the therapy goal

**Fig. 3:**
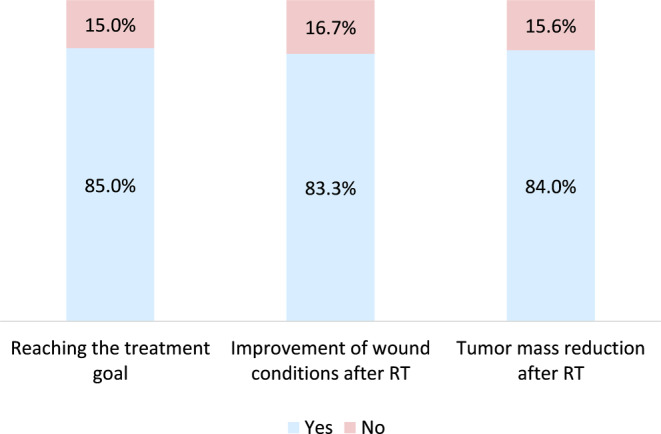
Bar chart showing the achievement of the therapy goal, improvement of wound conditions and tumor mass reduction after RT

Overall, the treatment goal was to treat ulceration in 98 patients and to prevent ulceration in three patients. The treatment goal was achieved as described in Sect. 2.1 in 85 patients (84.2%, Fig. 3. It was not achieved in 15 patients (14.9%), one patient (0.9%) was lost to follow-up (Table 3).

**Table 3:** Patient outcomes after current radiotherapy.

Outcome	*n* (%)	Median (min–max)
*Recurrence/progression, any*	53 (52.5)	–
Local recurrence/progression	31 (30.7)	–
Distant metastases	25 (24.8)	–
Progression of distant metastases	8 (7.9)	–
*No recurrence or progression*	27 (26.7)	–
*Lost to follow-up*	21 (20.8)	–
*Re-radiation*	16 (15.8)	–
Same location, (applied dose (Gy))	5 (5.0)	23.4 (8.8–40)
Different location	11 (10.9)	–
*Overall survival (months)*	–	8 (0–202)
*Time to progression (months)*	–	4 (0–112)
*Progression-free survival (months)*	–	5 (0–202)

**Fig. 4:**
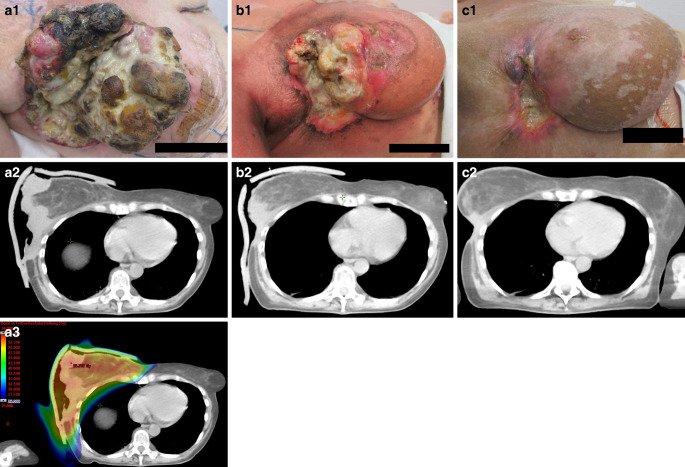
**a–c** Example of a patient with a malignant wound responding well to RT (tumor mass reduction and improvement in wound conditions) and achieving the treatment goal (Breast carcinoma, invasive carcinoma without special type, grade 3, triple negative, T4b N1 M1, combined radio-chemotherapy with vinorelbine). (*a1*) Wound documentation before RCT, (*a2*) axial slice of the pre-treatment RT planning CT scan, (*a3*) corresponding VMAT radiotherapy plan, dose color wash ranging from 25.0 Gy (blue, lowest value) to maximum dose (55.757 Gy, red, base plan) on this plane, (*b1–2*) wound documentation and CT scan at the end of RCT (total dose: 66.6 Gy/1.8 Gy/fraction) and (*c1–2*) 2 months after RCT showing treatment response

**Fig. 5:**
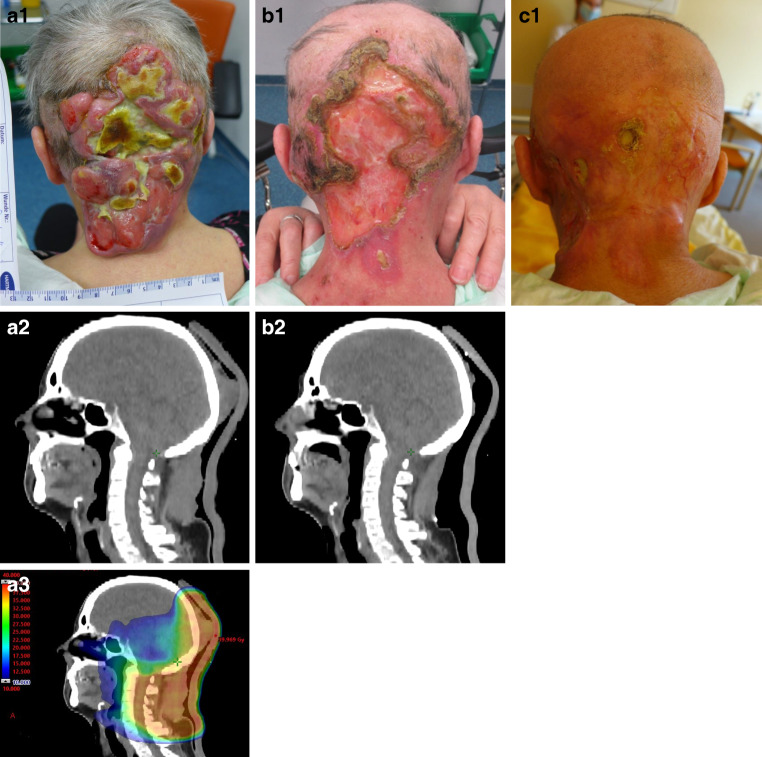
**a–c** Example of patient with malignant wound responding well to RT (tumor mass reduction and improvement in wound conditions) and achieving the treatment goal: (Follicular B‑cell lymphoma, initial stage IIIB, palliative RT). (*a1*) Wound documentation before RT, (*a2*) axial slice of the pre-treatment RT planning CT, (*a3*) VMAT-radiotherapy plan, dose color wash ranging from 10 Gy (blue, lowest value) to 39,969 Gy (maximum dose, red) on this plane. (*b1–2*) Wound documentation and CT-scan at the end of RT and (*c*) wound documentation 3.5 months after RT

Of the 15 patients who did not reach their treatment goal, four discontinued RT before receiving 80% of the planned dose. Two patients experienced local tumor progression during RT, one patient suffered from an increase in ulceration during RT, one patient showed progression one week after completing RT. In the remaining seven patients, RT did not lead to symptom improvement (such as pain reduction or hemostasis), tumor mass reduction or wound improvement. Supplemental Table 2 comprises details concerning the 11 patients which did not achieve the predefined therapy goal despite receiving ≥ 80% of the prescribed RT dose. Refer to Figs. 4 and 5 for examples of a good response to RT for malignant wounds Supplemental Fig. 1 for an example of a patient not reaching the treatment goal.

The correlation between independent variables and the achievement of the therapy goal was analyzed using univariable logistic regression. Herein, the application of concomitant systemic therapy was statistically significantly associated with a more frequent achievement of the therapy goal (Table 4).

**Table 4:** Influence of independent variables on the achieving of the therapy goal.

Variable	Achieving the therapy goal	
	Hazard ratio (95% CI)	*P*-value univariable
*Age, per year*	1.02 (0.98–1.06)	0.26
*Gender*		
Female (*n* = 66) versus male (*n* = 35)	1.29 (0.42–3.97)	0.66
*BMI, per kg/m*^*2*^	1.03 (0.91–1.16)	0.64
*Charlson Comorbidity Index, per point*	1.00 (0.78–1.26)	0.98
*EQD2 (a/ß:10), per Gy*	1.03 (1.00–1.07)	0.08
*Single dose, per Gy*	0.91 (0.54–1.56)	0.74
*Flap use*		
No (*n* = 29) versus yes (*n* = 67)	0.75 (0.23–2.45)	0.63
*Concomitant systemic therapy*		
Yes (*n* = 33) versus no (*n* = 68)	8.45 (1.06–67.37)	**0.044**
*Radiation technique*		
Electrons (*n* = 12) versus photons (*n* = 84)	2.01 (0.24–17.00)	0.52
*Tumor entities*		
Squamous cell carcinoma of the skin (*n* = 23) versus other (*n* = 78)	2.05 (0.65–6.50)	0.22
Breast carcinoma (*n* = 27) versus other (*n* = 74)	0.66 (0.20–2.14)	0.49
*Antibiotic therapy*		
Yes (*n* = 18) versus no (*n* = 83)	1.51 (0.31–7.35)	0.61

Logistic regression analysis was used for calculations. *P* values < 0.05 were considered to be statistically significant

*CI* confidence interval, *BMI* Body mass index, *Gy* Gray

### Overall survival

**Fig. 6:**
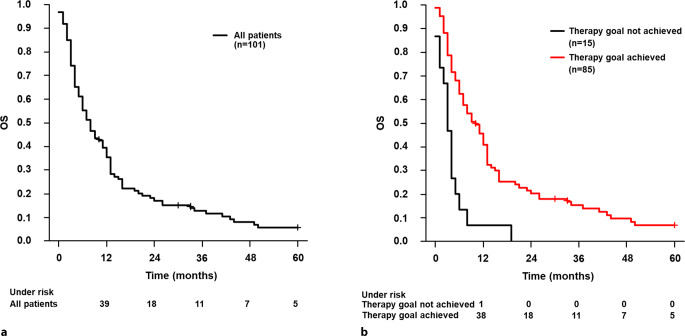
**a** Kaplan-Meier curve for overall survival of all patients. **b** Kaplan-Meier curve on overall survival after the start of radiotherapy depending on the achievement of the treatment goal (log-rank *p* < 0.01). *OS* overall survival

Median overall survival (OS) was 8 months from the start of RT, ranging from 0 to 202 months. 39.5% of patients were alive at 12 months, 17.2% at 24 months and 12.9% at 36 months (Fig. 6). A total of 97 patients (96%) died during the observation period. The influence of different variables on OS was analyzed using univariable and multivariable Cox regression.

In the univariable analysis, CCI, the application of concomitant systemic therapy and the achievement of the therapy goal proved to be significant. In multivariable Cox regression, these factors remained statistically significant: CCI of over 7 was associated with a higher risk of death (*p* = 0.018, HR = 1.661, 95% CI: 1.092–2.526). Achieving the treatment goal (*p* < 0.001, HR = 0.334, 95% CI: 0.183–0.610) and concomitant systemic therapy (*p* = 0.037, HR = 0.608, 95% CI: 0.380–0.970) were significantly correlated with an improved OS (Fig. 6; Table 5).

**Table 5:** Influence of variables on the overall survival of patients. Calculations were performed using univariable Cox regression.

Variable	Univariable		Multivariable	
	Hazard ratio (95% CI)	*P*-value	Hazard ratio (95% CI)	*P*-value
*Age, in years*				
≥ 70 (*n* = 53) versus < 70 (*n* = 48)	1.34 (0.89–2.03)	0.16	n. a.	n. a.
Continuous	1.01 (0.99–1.03)	0.07	n. a.	n. a.
*Sex*				
Female (*n* = 66) versus male (*n* = 35)	0.90 (0.59–1.38)	0.63	n. a.	n. a.
*BMI (kg/m*^*2*^*)*				
≥ 25 (*n* = 31) versus < 25 (*n* = 49)	0.71 (0.45–1.13)	0.15	n. a.	n. a.
≥ 30 (*n* = 12) versus < 30 (*n* = 68)	1.06 (0.56–2.01)	0.87	n. a.	n. a.
*Charlson comorbidity index, in points*				
≥ 7 (*n* = 59) versus < 7 (*n* = 42)	1.53 (1.01–2.31)	**0.044**	1.66 (1.09–2.53)	**0.018**
*Total dose, in Gy*				
≥ 45 (*n* = 54) versus < 45 (*n* = 47)	0.71 (0.47–1.06)	0.09	n. a.	n. a.
Continuous	0.99 (0.98–1.00)	0.09	n. a.	n. a.
*Single dose, in Gy*				
> 2 (*n* = 33) versus ≤ 2 (*n* = 68)	1.27 (0.82–1.96)	0.29	n. a.	n. a.
*Radiation technique*				
Electrons (*n* = 12) versus photons (*n* = 84)	0.97 (0.52–1.78)	0.91	n. a.	n. a.
*Flap use*				
No (*n* = 29) versus yes (*n* = 67)	0.88 (0.56–1.39)	0.59	n. a.	n. a.
*Concomitant systemic therapy*				
Yes (*n* = 33) versus no (*n* = 68)	0.55 (0.35–0.87)	**0.011**	0.611 (0.38–0.97)	**0.037**
*Achievement of the therapy goal*				
Yes (*n* = 85) versus no (*n* = 15)	0.31 (0.18–0.56)	**<** **0.001**	0.33 (0.18–0.61)	**<** **0.001**
*Tumor entities*				
Cutaneus squamous cell carcinoma (*n* = 23) versus other (*n* = 78)	1.23 (0.82–1.84)	0.32	n. a.	n. a.
Breast carcinoma (*n* = 27) versus other (*n* = 74)	0.82 (0.52–1.29)	0.39	n. a.	n. a.
*Antibiotic therapy*				
Yes (*n* = 18) versus no (*n* = 83)	0.79 (0.46–1.36)	0.40	n. a.	n. a.

*P*-values < 0.05 were considered to be statistically significant. The variables with statistically significant *p*-values (in bold) in univariable analysis were tested sequentially in multivariable Cox regression

*CI* confidence interval, *Gy* Gray, *n.* *a.* not applicable

A subgroup analysis was performed with regard to overall survival according to the use of concomitant systemic therapy. Patients were divided into curative and palliative subgroups. In patients with curative intent, concomitant systemic therapy had a significant impact on overall survival (log-rank *p* = 0.036).

## Discussion

In this retrospective analysis of 101 patients with MFW treated with RT, we observed high rates of clinical benefit. A total of 85% of patients achieved the predefined therapeutic goal, over 80% experienced tumor reduction and symptom improvement, and RT-related toxicity was generally mild. Concurrent chemotherapy and a lower CCI were significantly associated with improved outcomes and OS. Despite the heterogeneity of tumor types, treatment intent, and RT regimens, our results underscore the potential of RT—either alone or in combination with systemic therapy—for local tumor control and symptom relief in patients with MFW.

### Patient characteristics

The most frequent tumor entities in our cohort were breast cancer, cutaneous squamous cell carcinoma, and vulvar cancer. This distribution is consistent with previous studies, such as Kondra et al., who reported a predominance of breast cancer (55%), SCC (25%), and sarcoma (9%) in a similar patient population [17]. Maida et al. likewise found breast cancer to be the most common cause of MFW, followed by gastrointestinal and lung cancers [5]. In our cohort, the most common anatomical locations were the head and neck, followed by the breast/chest wall and genitals—findings that are comparable to earlier reports. A majority of patients (65.3%) were female, reflecting the high incidence of breast cancer-associated MFW. This gender distribution aligns with existing literature on malignant wounds [5, 17, 18].

### Treatment outcome

The achievement of a treatment-related therapy goal, as defined in Sect. 2.2, was observed in 85% of patients. Our composite endpoint aimed to incorporate both objective parameters (e.g., Haisfield-Wolfe and Baxendale-Cox wound stages [15]) and subjective outcomes (e.g., pain relief), thus enabling a structured yet clinically relevant assessment in both curative and palliative settings. Nevertheless, the use of such a surrogate endpoint introduces interpretation limitations and reduces comparability with other studies. Generally, comparisons with the literature are hampered by the limited number of studies on RT in MFW, particularly outside of breast cancer. Nakamura et al. conducted a prospective study on 21 patients with breast cancer and skin invasion, demonstrating significant symptom relief but no improvement in pain or quality of life (QoL), possibly due to low baseline pain levels and disease progression outside the RT field [12]. Chia et al. reported symptom improvement in 94% of patients with ulcerated breast cancer, with a partial remission in 46% and stable disease in 48% [13]. In our study, local progression or recurrence occurred in 30.7% of patients, with a median time to progression of 4 months—somewhat shorter than the 6–10 months reported by Chia et al. and Nakamura et al. [12, 13].

Concomitant chemotherapy significantly increased the likelihood of achieving the therapy goal (*p* = 0.044). The benefit of combining RT and chemotherapy has been previously demonstrated for local tumor response and OS, particularly in patients with good performance status [19, 20]. In our cohort, RCT was more frequently used in curative settings (41.9%) than in palliative ones (10%), reflecting individualized treatment decisions tailored to patient condition and treatment intent. Although some studies report increased toxicity with RCT, this was not observed in our cohort—potentially due to careful patient selection and heterogeneous regimens [21]. These findings support a context-dependent use of concurrent RCT in both curative and palliative scenarios [22].

RT-related toxicity was generally mild: 73.3% of patients experienced any adverse event, with grade 1–2 dermatitis occurring in 58.6% and grade 3 dermatitis in 6.9%. Only two patients discontinued RT due to toxicity. These results are comparable to previous findings, such as those of Jacobson et al., who reported grade 1–2 dermatitis in 90% of patients, and Chia et al., who documented no grade ≥ 3 toxicities [10, 13]. Similarly, Nakamura et al. reported grade 2 dermatitis in one patient and grade 3 in two patients (10%) [12]. Prior RT at the same site did not appear to increase toxicity in our cohort, consistent with the findings of Jacobson et al. [10].

Interestingly, total dose and fractionation did not correlate with therapy goal achievement or OS. Most patients (59.4%) received normofractionated RT with 1.8–2 Gy per fraction, even in palliative settings. Hypofractionated schemes were more frequently used in recent years, reflecting evolving practice patterns and recommendations favoring shorter courses in patients with limited life expectancy [22–24]. Among non-responders, seven patients showed no clinical benefit, while four experienced disease progression despite initial response. The relatively high local recurrence rate (30.7%) underscores the aggressive nature of MFW and the importance of tailored treatment strategies.

### Survival

Median OS in our cohort was 7.8 months from the start of RT, with 39.5% of patients alive at 12 months. Achievement of the therapy goal, concurrent chemotherapy, and lower CCI were all associated with improved survival. These findings are consistent with the established prognostic value of comorbidity burden and suggest that local tumor control—captured here through goal achievement—may reduce morbidity and, indirectly, mortality. While survival benefits from concurrent chemotherapy were evident primarily in curatively treated patients, the data support its potential use in selected palliative cases as well [21, 25].

### Limitations and conclusions

This retrospective study analyzed a cohort of 101 patients with MFW treated with RT at a tertiary academic oncology center between January 2000 and June 2022. We evaluated the achievement of predefined therapeutic goals, wound improvement, oncological outcomes, and factors influencing treatment success. The main limitations of this study include its retrospective design, the heterogeneity of primary tumor types, and variability in treatment regimens, including different fractionation schedules and RT techniques. In addition, QoL data were not systematically collected.

Despite these limitations, the primary treatment goal—defined as reduction of tumor burden, bleeding control, pain relief, and overall wound improvement—was achieved in 85% of patients, with acceptably low toxicity. Concurrent chemotherapy was significantly associated with goal achievement and, along with a lower CCI, was also linked to improved overall survival.

To our knowledge, this is the largest cohort of patients with MFW treated with RT reported to date. These findings highlight the relevance of RT as an effective component of multimodal management strategies in both curative and palliative settings, contributing to local tumor control and potentially improving patients’ QoL.

## Supplementary Information

Suppl. Table 1: Adverse events according to Common Terminology Criteria for Adverse Events. Suppl. Table 2: Patients who did not reach the treatment goal despite receiving ≥ 80% of the prescribed RT dose. Suppl. Figure 1: Example of a patient who did not achieve the treatment goal

## Data Availability

The datasets generated and/or analyzed in the current study are available from the corresponding author upon reasonable request.
