# Supplementary material for: Radiotherapy in the treatment of malignant fungating wounds: clinical practice, response rates, and outcome from a tertiary cancer center
Source: Strahlenther Onkol. 2025 Aug 12;202(5):485–95. doi: 10.1007/s00066-025-02443-7 (PMC13109203; doi:10.1007/s00066-025-02443-7)
Supplement: Supplementary file 1 — Suppl. Table 1: Adverse events according to Common Terminology Criteria for Adverse Events. Suppl. Table 2: Patients who did not reach the treatment goal despite receiving ≥ 80% of the prescribed RT dose. Suppl. Figure 1: Example of a patient who did not achieve the treatment goal [file 66_2025_2443_MOESM1_ESM.pdf]

Supplemental Material

Radiotherapy in the treatment of malignant fungating wounds: clinical practice, response rates, and outcome from a tertiary cancer center

Anna Lena Reinking[1], Martin Leu[1], Leif Hendrik Dröge[1], Benedikt Kieslich[1], Sandra Donath[1], Markus Anton Schirmer[1,4], Stephanie Bendrich[1], Laura Anna Fischer[1], David Alexander Ziegler[1,3], Hannes Treiber[2], Enver Aydilek[2], Raphael Koch[2], Stefan Rieken[1] and Manuel Guhlich[1,*]

[1] Clinic of Radiotherapy and Radiation Oncology, University Medical Center Göttingen, Göttingen, Germany

[2] Department of Hematology and Medical Oncology, University Medical Center Göttingen, Göttingen, Germany

[3] Clinic of Radiation Oncology and Radiotherapy, Medical University Lausitz – Carl Thiem, Cottbus, Germany

[4] Quality Conferences Office at the Clinical State Registry Baden-Württemberg GmbH, Baden-Württemberg Cancer Registry (BWCR), Stuttgart, Germany

[*] Correspondence:
manuel.guhlich@med.uni-goettingen.de; Tel.: +49 551 39 64505
Orcid-ID: 0000-0002-2599-6196

Supplemental Table 1: Adverse events were graded according to Common Terminology Criteria for Adverse Events (Version 5.0) [16]. *Number of patients whose worst adverse event was grade 1, 2, or 3. °For n=20 patients not analyzed due to concomitant chemotherapy, for n=2 patients not analyzed due to Hemoglobin-relevant tumor bleeding, for n=3 the scoring was inconclusive considering the dose and PTV volume applied. #vaginal mucositis as scored by CTCAE Version 3. There were no adverse events exceeding grade 3.

**Adverse events (acute & late)**	**Grade 1: n (%)**	**Grade 2: n (%)**	**Grade 3: n (%)**
Any adverse event*	28 (27.7)	35 (34.7)	11 (10.9)
Radiation Dermatitis	25 (24.8)	34 (33.7)	7 (6.9)
Mucositis oral	7 (6.9)	5 (5.0)	1 (1.0)
White blood cell decreased (n=57°)	2 (3.5)	1 (1.8)	1 (1.8)
Platelet count decreased (n=57°)	1 (1.8)	0 (0)	1 (1.8)
Cystitis	1 (1.0)	0 (0)	1 (1.0)
Thrush	0 (0)	5 (5.0)	0 (0)
Dry mouth	7 (6.9)	2 (2.0)	0 (0)
Proctitis	5 (5.0)	1 (1.0)	0 (0)
Anemia (n=57°)	4 (7.0)	1 (1.8)	0 (0)
Diarrhea	4 (4.0)	1 (1.0)	0 (0)
Skin hyperpigmentation	10 (9.9)	0 (0)	_
Nausea	7 (6.9)	0 (0)	0 (0)
Dysphagia	5 (5.0)	0 (0)	0 (0)
Lymphedema	4 (4.0)	0 (0)	0 (0)
Conjunctivitis	3 (3.0)	0 (0)	0 (0)
Dysgeusia	3 (3.0)	0 (0)	_
Skin hypopigmentation	2 (2.0)	0 (0)	_
Urticaria	1 (1.0)	0 (0)	0 (0)
Lokalized edema	1 (1.0)	0 (0)	0 (0)
Periorbital edema	1 (1.0)	0 (0)	0 (0)
Superficial soft tissue fibrosis	1 (1.0)	0 (0)	0 (0)
Vaginal mucositis#	1 (1.0)	0 (0)	0 (0)

Supplemental Table 2: Patients who did not reach the treatment goal despite receiving ≥ 80% of the prescribed RT dose, *total dose without previous dose, ° OS from the start of radiotherapy. None of the patients received concomitant chemotherapy. RT = radiotherapy, VMAT = volumetric modulated arc therapy, IMRT = intensity modulated RT, 3DcRT = 3D conformal RT, EQD2 = equivalent dose in 2-Gy fractions, cm = centimetre.

Patient	Tumor entity	Location	MFW underlying tumor manifestation	Recurrence	Prior RT at the same location	Therapy concept	RT technique	Total dose *	Fractionation Fx * (Gy)	EQD2 (α/β=10)	Flap (cm)	Reasons for not achieving the therapy goal; achievements, if applicable	OS° (months)
#1	Cutaneous SCC	Head/ Neck	Primary tumor	no	no	definite RT	IMRT	64.0	32 * 2.0	64.00	1	Progress 1 week after the end of RT; improvement in wound conditions	3
#2	Cutaneous SCC	Extremities	Primary tumor	no	no	definite RT	3DcRT	59.4	33 * 1.8	58.41	1	stable disease	2
#3	Cutaneous SCC	Extremities	Metastasis	yes	no	palliative RT	3DcRT	55.0	22* 2.5	57.29	1	stable disease	4
#4	Breast cancer	Back	Metastasis	yes	yes, 30 Gy	palliative RT	Brachytherapy	39.0	13* 3.0	42.25	no	Increase in ulceration; tumour mass and pain reduction	4
#5	Breast cancer	Breast/ chest wall	Primary tumor	no	no	palliative RT	3DcRT	45.0	15* 3.0	48.75	1	stable disease	3
#6	Osteosarcoma	Breast/ chest wall	Metastasis	yes	yes, 60 Gy	palliative RT	VMAT	45.0	15* 3.0	48.75	1	Progress under RT; bleeding stop	4
#7	Breast cancer	Breast/ chest wall	Metastasis	no	no	palliative RT	3DcRT	30.0	10 * 3.0	32.50	no	stable disease	1
#8	Rectal carcinoma	Abdomen	Locoregional lymph node metastasis	yes	no	palliative RT	IMRT	45.0	15* 3.0	48.75	1	stable disease	6
#9	Bronchial carcinoma	Breast/ chest wall	Metastasis	no	yes, 40 Gy	palliative RT	3DcRT	30.0	15* 2.0	30.00	no	stable disease	8
#10	Pleural mesothelioma	Head/ Neck	Metastasis	no	no	palliative RT	VMAT	39.0	13* 3.0	42.25	1	stable disease	1
#11	Dermal sarcoma	Head/ Neck	Primary tumor	yes	no	definite RT	IMRT	66.0	33 * 2.0	66.00	1	Progress under RT; improvement in wound conditions, stop of bleeding and secretion	19

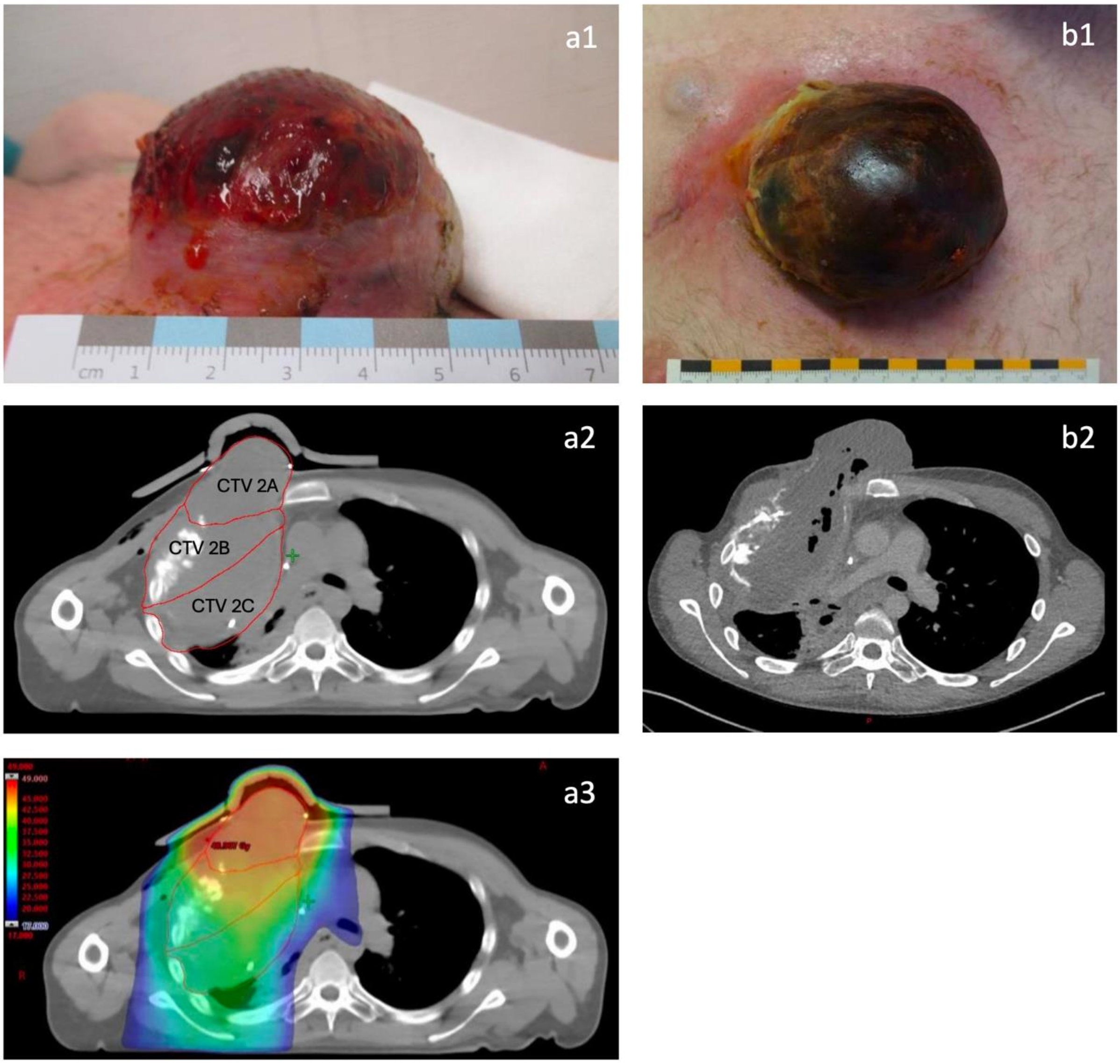

Supplemental Figure 1: Example of a patient who did not achieve the treatment goal (Osteosarcoma of osteoblastic type, initial T2 N0 M0, grade 3, previous RT of 60 Gy to the same site, divided into CTV2A-C due to previous RT). (a1) Wound documentation before RT, (a2) axial slice of the pre-treatment RT planning CT (a3) VMAT-RT plan, dose color wash ranging from 17 Gy (blue, lowest value) to 48,907 Gy (maximum dose, red) on this plane. (b1-2) Wound documentation and CT-scan at the end of RT: although the tumor bleeding stopped, pulmonary metastasis progressed, therefore, treatment goal was accounted as “not achieved”.
